# Efficacy and complications associated with a modified inferior alveolar nerve block technique. A randomized, triple-blind clinical trial

**DOI:** 10.4317/medoral.19554

**Published:** 2014-03-08

**Authors:** Marta Montserrat-Bosch, Rui Figueiredo, Pedro Nogueira-Magalhães, Josep Arnabat-Dominguez, Eduard Valmaseda-Castellón, Cosme Gay-Escoda

**Affiliations:** 1 DDS. Resident of the Master degree program in Oral Surgery and Implantology. Faculty of Dentistry – University of Barcelona; 2 DDS, PhD. Master degree program in Oral Surgery and Implantology. Associate professor of Oral Surgery and Professor of the Master degree program of Oral Surgery and Implantology. Faculty of Dentistry - University of Barcelona. Researcher of the IDIBELL institute; 3 MD, DDS, PhD. Master degree program in Oral Surgery and Implantology. Associate professor of Oral Surgery. Faculty of Dentistry - University of Barcelona. Researcher of the IDIBELL institute; 4 DDS, PhD. Master degree program in Oral Surgery and Implantology. Professor of Oral Surgery and professor of the Master degree program of Oral Surgery and Implantology. Faculty of Dentistry – University of Barcelona. Researcher of the IDIBELL institute; 5 MD, DDS, PhD. Chairman and Professor of Oral and Maxillofacial Surgery Department. Director of the Master of Oral Surgery and Implantology. Faculty of Dentistry - University of Barcelona. Coordinating investigator of the IDIBELL Institute. Head of the Service of Oral and Maxillofacial Surgery, Teknon Medical Center. Barcelona, Spain

## Abstract

Objectives: To compare the efficacy and complication rates of two different techniques for inferior alveolar nerve blocks (IANB). Study Design: A randomized, triple-blind clinical trial comprising 109 patients who required lower third molar removal was performed. In the control group, all patients received an IANB using the conventional Halsted technique, whereas in the experimental group, a modified technique using a more inferior injection point was performed. Results: A total of 100 patients were randomized. The modified technique group showed a significantly higher onset time in the lower lip and chin area, and was frequently associated to a lingual electric discharge sensation. Three failures were recorded, 2 of them in the experimental group. No relevant local or systemic complications were registered. Conclusions: Both IANB techniques used in this trial are suitable for lower third molar removal. However, performing an inferior alveolar nerve block in a more inferior position (modified technique) extends the onset time, does not seem to reduce the risk of intravascular injections and might increase the risk of lingual nerve injuries.

** Key words:**Dental anesthesia, inferior alveolar nerve block, lidocaine, third molar, intravascular injection.

## Introduction

Pain control is one of the main concerns to both dentists and patients during dental treatments. Therefore many reports have been published on this topic (1-6). Nevertheless, there are still some situations where dental healthcare professionals are not able to attain an adequate anesthesia (7-10). Traditionally, pain management in the mandible, especially in the molar region, is far more complex than in the maxilla. Several anatomical factors like the presence of a thick bone cortical plate, the thickness of soft tissue through which the needle must penetrate and the possibility of accessory innervations have been related to the low efficacy of inferior alveolar nerve blocks (IANB) (8). However, most authors explain the high failure rates (up to 15-20%) associated with IANB with a deficient technique, due to the difficulty of accurately locating the neurovascular bundle (11). Another important disadvantage of IANB is the high risk of intravascular injections, which can lead to systemic complications (12).

**Figure 1:**
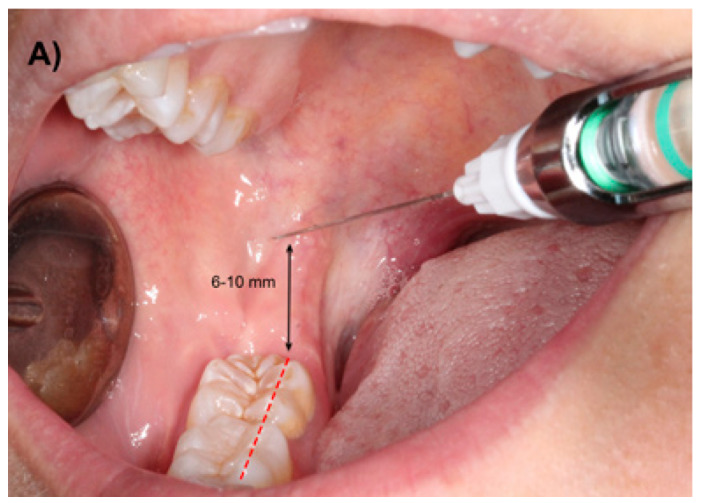
A) Conventional IANB using the technique described by Halsted.

The most commonly used IANB technique is the Halsted approach or direct technique. The target point for the deposition of the local anesthetic is the inferior alveolar nerve (IAN) before it enters the mandibular foramen. The site of needle penetration is the mucous membrane on the medial side of the mandibular ramus. With the patient placed in a supine or semisupine position, with his mouth wide open, the index finger or thumb should be placed in the coronoid notch. A horizontal line that extends posteriorly from the fingertip in the coronoid notch to the deepest part of the pterygomandibular raphe should be imagined. This line should be parallel and, in most patients lies 6 to 10 mm above the occlusal plane of the mandibular molars (Fig. 1). The needle insertion point lies three-quarters of the anteriorposterior distance from the coronoid notch to the deepest part of the pterygomandibular raphe. The barrel of the syringe should be placed in the contralateral side of the mouth, usually over the premolars (13), and the needle should be inserted approximately 25mm until bone contact is noted.

This approach has a considerable rate of positive aspirations, which indicates that systemic alterations may be frequent due to possible intravascular injection of the local anesthetic (14-16). A study published in our department found an incidence of positive aspiration of 8.9% (15). However, the same report stressed that several factors like the syringe model might influence this figure (15,16).

**Figure 2:**
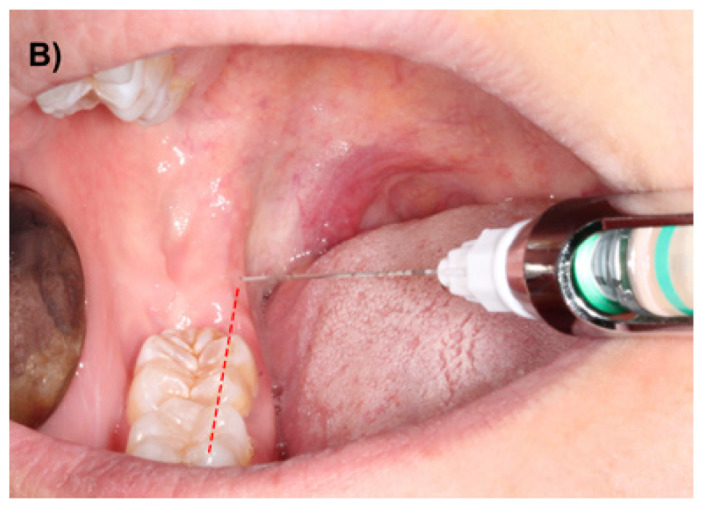
B) Modified IANB using a lower injection location.

A possible way to decrease the incidence of these events is to change the injection site to a more inferior position, since the needle tip would be in a location where the neurovascular bundle would be inside the mandibular canal, therefore avoiding direct contact with the vascular structures. Thus, using a modified Halsted technique with a slightly inferior injection height (at the occlusal plane level) would probably reduce positive aspirations without significantly compromising the efficacy of IANB (Fig. 2). For this reason, the authors decided to perform a clinical trial with the aims of comparing the efficacy and the complication rates of these two IANB techniques.

## Patient and Methods

A randomized, triple blind clinical trial was performed in 109 patients. All participants were submitted to the surgical removal of an impacted lower third molar between the January and June 2012 in the Dental Hospital of the University of Barcelona. This study was designed complying with the CONSORT guidelines for clinical trials (17).

The study was approved by the Research Ethics Committee (CEIC) of the Dental Hospital of the University of Barcelona. Before enrolment, all patients were explained the objectives, implications and possible complications of this clinical trial and agreed to participate by signing an informed consent. The patients didn’t receive any financial compensation for their participation in the study. The Helsinki declaration guidelines for research have been followed. The main inclusion criterion was the presence of an impacted lower third molar that required surgical removal. Exclusion criteria were patients aged below 18 years or over 60 years, patients with significant systemic diseases (classification ASA III or ASA IV), pregnancy and breast feeding, history of allergy to lidocaine or other dental anesthetics, cardiovascular pathology that contraindicates the administration of local anesthetic with vasoconstrictor, bleeding disorders, patients under anticoagulant therapy, presence of trauma or symptoms associated to the third molar 30 days prior to extraction, history of analgesic and/or anti-inflammatory drugs intake 7 days before surgery, significant pathology of adjacent teeth (lower first and second molar), third molars in ectopic positions and surgical procedures with an extraction time of over 60 minutes. Antibiotic prophylaxis was not performed. All surgeries were performed by third-year residents of the Master degree program of Oral Surgery and Implantology (University of Barcelona) using a similar surgical technique. The extraction of impacted lower third molars was performed under local anesthesia with lidocaine 2% and epinephrine 1:80.000 (Xylonibsa; Inibsa, Lliça de Vall, Spain). In the experimental group, a modified technique was used, where the injection point was located at the occlusal plane level about three-quarters of the distance from the anterior border of the ramus (Fig. 2). On the other hand, in the patients included in the control group, the conventional Halsted approach was employed (Fig. 1). In both groups, the needle was slightly withdrawn after contact with bone (1mm), aspiration was carried out, and if no blood was observed inside of the cartridge, the anesthetic solution was slowly injected. Approximately 1,3 ml of the solution was deposited in this area and the remaining 0,5 ml were infiltrated while extracting the needle in order to guarantee the anesthesia of the lingual nerve. The syringe used in both techniques was the UnijectTM® (Hoechst AG, Frankfurt, Germany) with a 35 mm long and 27G Monoprotect XL® needle (Inibsa, Lliça de Vall, Spain). In order to attain an adequate anesthesia to perform the third molar extraction, an additional infiltration of 1,8 ml of the anesthetic solution was done in the buccal region. Thermal sensibility tests of the homolateral lower first molar were made every 30 seconds by placing a cotton pellet with tetrafluoroethane (Endo Ice® Refrigerant Spray, Coltène/Waledent Gmbh+ Co. KG, Langenau, Germany) on the buccal aspect of the tooth until a negative result was obtained. The surgical field and all the surgical material were sterile. The surgeon raised a full-thickness flap, which was protected by the Minnesota retractor. A lingual flap retraction using a Freer periosteal elevator was only performed when the surgeon consider it to be necessary. Sterile low-speed (20.000 rpm) handpieces and sterile saline solution were used for bone removal and tooth sectioning when necessary. To close the wound, 3-0 silk sutures (Silkam, Braun; Tuttlingen, Germany) were used. The surgical tech-nique was similar to that described by Leonard (18).

The following variables were collected: age, gender, operated side, extraction time, number of cartridges used, third molar position according to the Pell & Gregory and Winter (19) classifications, flap design, need for bone removal and tooth sectioning, and the presence of blood inside of the cartridge after aspiration (positive aspiration). The surgeon also observed and explored the area of injection in order to detect possible local complications (hematoma, hemorrhage, trismus, lingual nerve and IAN injuries, infection, presence of ulcers, swelling, etc.) and registered any systemic alterations. Additionally, the patients filled a 100 mm visual analog scale (VAS) to measure pain intensity during injection and were asked if they noticed any electric discharge sensation in the lower lip or tongue areas. In order to estimate the efficacy of both techniques, the following data were gathered: onset time (time elapsed from the administration of the anesthetic to the presence of tingling sensation in the lower lip, chin and tongue regions), the need for an additional injection (using a different approach, i.e., infiltration, intraligamentous anesthesia, etc.), a thermal vitality test (applying a frozen cotton pellet with tetrafluoroethane on the buccal aspect of lower first molar), and the need for an additional IANB (absence of Vincent sign 5 minutes after injection, which was considered as a failure of the initial block). Pain during extraction was also measured through a 100 mm VAS.

The sample size was calculated using the software G* Power 3.0. (Heinrich-Heine-Universität, Düsseldorf, Germany).

The group assignment for each patient was predetermined by a sequence of random numbers in blocks (generated in www.randomization.com). The incorporation of each subject in the study was decided before knowing the assigned group (the researchers who assess patient eligibility did not have access to the randomization sequence).

After the randomization process, 2 third-year fellows of the Oral Surgery and Implantology Unit, performed all the IANB with both techniques. These surgeons did not gather any further data, to avoid compromising the blinding process. All patients, the statistician and the researchers responsible for collecting the data (onset time, presence of systemic complications, the need for additional injections and thermal vitality tests performed by the surgeons) were unaware of the applied technique. Although the surgeons who performed the extraction and the vitality tests were not informed of the patient group, in some cases, the blinding process might have been compromised due to the presence of a small bleeding point in the injection area.

Statistical analysis was performed using SPSS Software for Windows 15.0 (SPSS v15.0, SPSS Inc. Chicago, USA, license from the University of Barcelona). Normality of scale variables (patient age, extraction time, pain during injection and extraction, first molar vitality tests and onset times) was explored using the Kolmogorov-Smirnov test. Where normality was rejected, the interquartile range (IQR) and median were calculated. Where distribution was compatible with normality, the mean and standard deviation (SD) were used. Parametric and nonparametric tests (Pearson chi-square, Fisher exact tests and Mann-Whitney U-tests) were used to compare the groups. The level of significancy was set at *p*<0.05.

## Results

**Figure 3:**
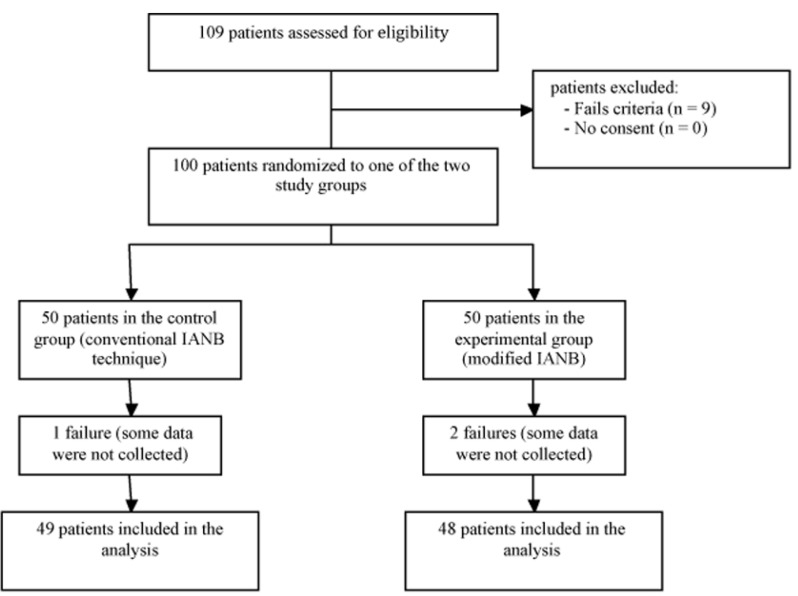
Flow diagram with the patients included in each stage of the trial.

A total of 100 patients with a mean age of 28.2 years (SD=8.4) were randomized (Fig. 3). Two patients in the experimental group and 1 patient in the control group did not refer a numbness area in the lower lip and chin region after 5 minutes. These patients received a second IANB and were considered as failures. Therefore, in these patients some variables were not gathered. The baseline and clinical characteristics for each group are shown in  Table 1. The variables related with complications and efficacy can be observed in  Table 2. Patients in the experimental group showed a significantly higher onset time in the lower lip and chin area (median time of 82.5 seconds vs. 45 seconds in the control group). An electrical discharge sensation in the tongue during injection was also significantly more frequent when the modified technique was used. However, no postoperative paresthesias were recorded. Additional injections (infiltrative, intraligamentous and intrapulpar) to obtain a more adequate pain control were less frequent in the control group (36.7% Vs. 47.9%; *p*>0.05).

**Table 1:**
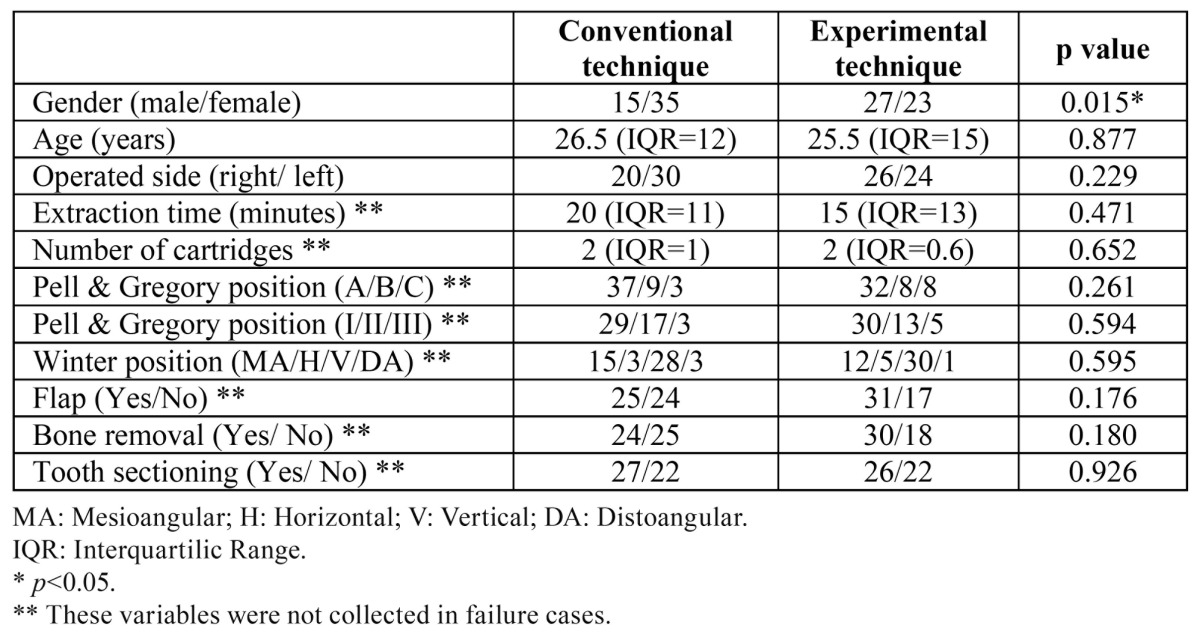
Baseline and clinical characteristics of the patients included in both groups.

**Table 2:**
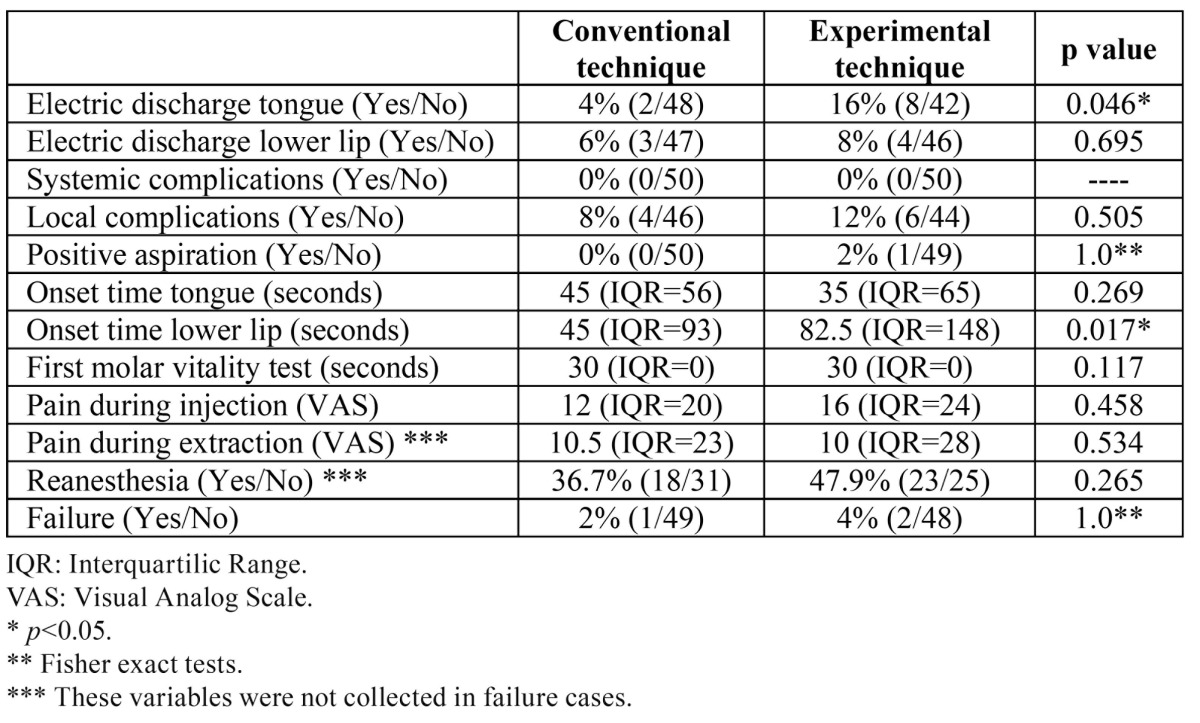
Complications and efficacy related variables of the patients included in both groups.

No important local complications (3 patients presented infection, 4 patients presented trismus, and 3 patients presented hematoma, trismus and swelling) or systemic complications were registered in this trial.

## Discussion

An adequate pain control when performing dental procedures in the posterior area of the mandible is often difficult to achieve. Infiltration techniques have shown extremely high success rates in the maxilla but seem to have disappointing figures when made in the lower molar region (6,20,21). Therefore, most authors recommend IANBs when a dental treatment is being made in this region (4,5,9,18). However, this technique can be difficult to perform specially because the anatomical landmarks used are not always reliable, and also because of the long distance between the injection point and the area where the local anesthetic is finally placed (5,7,13,22). This fact along with the considerably high positive aspiration rate (14-16) shows the need for alternative techniques to the traditional IANB. In the present report, a modified approach was tested with a more inferior injection location avoiding direct contact with the inferior alveolar blood vessels, in order to reduce the incidence of intravascular injections. This would, in theory, reduce the systemic effects of the anesthetic solution (12,22-24). However, in this trial, only one positive blood aspiration was recorded (1%), which didn’t allow an adequate comparison between both techniques regarding this variable. This outcome was quite surprising since a study made in our department showed a positive aspiration rate of 8.9% (15). The fact that in the previously mentioned report (15), the operators were instructed to perform 3 aspiration maneuvers during the injection (at the start, in the middle and at the end) while in this paper, only one aspiration was made (at the start) might explain this difference. This is one of the main limitations of the present paper, since the sample size calculation was made considering a higher incidence of positive aspirations. In order to avoid this limitation in future research, several aspirations should be made during injections and the sample size should be larger.

Lower third molar removal has been frequently considered as a good model in pain clinical trials (25,26). This surgical procedure requires a profound pulpal and soft tissue anesthesia and therefore, is suitable for a trial with these characteristics. Furthermore, several authors have thoroughly described this research design. In our opinion, the only problem that should be addressed in the future is related to the blinding of the surgeons who performed the extraction, since in some cases a small bleeding point can be observed in the injection area.

In the present report, an IANB was classified as a failure when the patient did not refer a numbness area in the lower lip and chin region after 5 minutes. Other authors, use longer times or the reanesthesia rate to assess the efficacy of IANB. Although no statistical significant differences were found between the 2 techniques regarding these variables ( Table 2), the experimental technique had a higher rate of failures (4% vs. 2%) and of reanesthesias (48% vs. 37%). All these results along with significantly longer onset time in the modified technique are probably related with the fact that the anesthetic solution is placed when the inferior alveolar nerve (IAN) is already inside of the mandibular canal. This fact might increase the onset time and slightly decrease the efficacy of IANB, since the local anesthetic needs more time reach the nerve, when compared to the conventional technique. These results clearly show that changing the injection site to a more inferior location, compromises the efficacy of IANBs.

Two cartridges of 2% lidocaine with epinephrine 1:80.000 were used in all the patients of our sample (1 cartridge in the IANB and another cartridge was infiltrated in buccal area). A meta-analysis published in 2011 (27) concluded that articaine solutions had a higher probability of achieving anesthetic success when compared with lidocaine (odds ratio of 2.44; 95% confidence interval: 1.59 to 3.76), especially when infiltration techniques are involved. Therefore, an improvement should also be expected when 4% articaine solutions are used.

One of the most severe local complications of IANB is the lesion of the lingual and inferior alveolar nerves (28). The fact that patients included in the experimental group frequently referred an electric discharge sensation in the tongue might indicate that the modified technique increases the incidence of lingual nerve impairments. A report by Pogrel and Thamby (29), which analyzed a pool of 83 patients with inferior alveolar nerve or lingual nerve injuries allegedly related to IANB, showed that 47 patients (56.6%) received a very painful injection or felt this electric shock sensation. Therefore, in our opinion, the injection location should be included in future studies about nerve injuries related to dental anesthesia, since it can be an important risk factor. If the patient refers an electric painful sensation during an IANB, the surgeon should avoid injecting the anesthetic solution in this area and should choose another injection location. Fortunately, these nerve lesions are extremely rare and the estimated incidence is almost neglectable (1 lesion for each 13.800.970 cartridges used) (30).

Both IANB techniques used in this trial are suitable for lower third molar removal, allowing an adequate pain management. However, performing an inferior alveolar nerve block in a more inferior position (modified technique) extends the onset time, does not seem to reduce the risk of intravascular injections and might increase the risk of lingual nerve injuries.
